# COVID-19 annual update: a narrative review

**DOI:** 10.1186/s40246-023-00515-2

**Published:** 2023-07-24

**Authors:** Michela Biancolella, Vito Luigi Colona, Lucio Luzzatto, Jessica Lee Watt, Giorgio Mattiuz, Silvestro G. Conticello, Naftali Kaminski, Ruty Mehrian-Shai, Albert I. Ko, Gregg S. Gonsalves, Vasilis Vasiliou, Giuseppe Novelli, Juergen K. V. Reichardt

**Affiliations:** 1 Department of Biology, Tor Vergata University of Rome, 00133 Rome, Italy; 2 Department of Biomedicine and Prevention, School of Medicine and Surgery, Tor Vergata University of Rome, Via Montpellier 1, 00133 Rome, Italy; 3 Department of Haematology and Blood Transfusion, Muhimbili University of Health and Allied Sciences, Dar es Salaam, Tanzania; 4 University of Florence, 50121 Florence, Italy; 5 College of Public Health, Medical and Veterinary Sciences, James Cook University, Smithfield, QLD 4878 Australia; 6 Core Research Laboratory, Istituto per lo Studio, la Prevenzione e la Rete Oncologica (ISPRO), Florence, Italy; 7 Institute of Clinical Physiology - National Council of Research (IFC-CNR), 56124 Pisa, Italy; 8 Pulmonary, Critical Care and Sleep Medicine, Yale School of Medicine, New Haven, CT USA; 9 Pediatric Hemato-Oncology, Edmond and Lilly Safra Children’s Hospital, Sheba Medical Center, Tel Hashomer 2 Sheba Road, 52621 Ramat Gan, Israel; 10 Department of Epidemiology of Microbial Diseases, Yale School of Public Health, New Haven, USA; 11 Instituto Gonçalo MonizFundação Oswaldo Cruz, Salvador, Bahia Brazil; 12 Department of Epidemiology of Microbial Diseases, Yale School of Public Health, New Haven, CT USA; 13 Department of Environmental Health Sciences, School of Public Health, Yale University, New Haven, USA; 14 IRCCS Neuromed, 86077 Pozzilli, IS Italy; 15 Department of Pharmacology, School of Medicine, University of Nevada, 89557 Reno, NV USA; 16 Australian Institute of Tropical Health and Medicine, James Cook University, Smithfield, QLD 4878 Australia

**Keywords:** Coronavirus, SARS-CoV-2, COVID-19, Pandemic, Susceptibility genes, Variants, Vaccines, Therapy, Public health

## Abstract

Three and a half years after the pandemic outbreak, now that WHO has formally declared that the emergency is over, COVID-19 is still a significant global issue. Here, we focus on recent developments in genetic and genomic research on COVID-19, and we give an outlook on state-of-the-art therapeutical approaches, as the pandemic is gradually transitioning to an endemic situation. The sequencing and characterization of rare alleles in different populations has made it possible to identify numerous genes that affect either susceptibility to COVID-19 or the severity of the disease. These findings provide a beginning to new avenues and pan-ethnic therapeutic approaches, as well as to potential genetic screening protocols. The causative virus, SARS-CoV-2, is still in the spotlight, but novel threatening virus could appear anywhere at any time. Therefore, continued vigilance and further research is warranted. We also note emphatically that to prevent future pandemics and other world-wide health crises, it is imperative to capitalize on what we have learnt from COVID-19: specifically, regarding its origins, the world’s response, and insufficient preparedness. This requires unprecedented international collaboration and timely data sharing for the coordination of effective response and the rapid implementation of containment measures.

## Introduction

Three and a half years after the start of the pandemic and the hoped-for endemic transition, the rush of severe acute respiratory syndrome coronavirus 2 (SARS-CoV-2) does not seem to have stopped, despite the slowdown in mortality facilitated by the massive global vaccination campaign [1]. Following the declaration of the end of the health emergency state [2], we are witnessing a world-wide reduction of preventive restrictions, a recovery of movements comparable to those of the pre-pandemic period and an increasingly forced coexistence with the virus. The past months have been characterized by the emergence of numerous descendent lineages of the Omicron BA.2 and BA.5 variants, *i.e.,* BQ.1, BQ.1.1, BA.4.6, BA.2.75.2, BF.7, and the recombinant XBB [3] and its subvariants including XBB1.5, which lately (as of June 2023) represents the most frequent among the sequences reported to Global Initiative on Sharing All Influenza Data (GISAID) [4–6]. Given the continuous evolution of the variants landscape, on March 15th, 2023, WHO has changed the monitoring and definition criteria, causing the de-escalation of the well-known BA.2, BA.4, and BA.5 from the list of Variants Of Concern (VOCs).

The efforts of the scientific community have led to a greater knowledge on the evolution of the virus, its pathogenic and molecular mechanisms; to a growing awareness of the symptoms, new therapeutic approaches for the treatment of the primary infection and of long-term effects (*i.e.,* Long COVID) [1], as well as the development of next-generation mucosal vaccines that could provide the basis for countering other respiratory viruses [7].

### Actual and potential evolution of SARS-CoV-2

The SARS-CoV-2 pandemic is the first contemporary disease for which we have gained substantial information on the dynamics of viral evolution and how this shapes the interaction with the host. Other viral outbreaks have riddled the twenty-first century, but the sheer amount of data accumulated, in geographical and temporal frames, provides a trove of information that will prove essential in dissecting pathogen–host interactions for years to come.

In particular, the frequent recourse to sequencing of the viral genome has given scientists an in-depth view on the stepwise evolution of the virus [6, 8].

Viruses are known for their high evolutionary rates due to rapid accumulation of nucleotide changes stemming from both intrinsic errors and viral-host cell interaction, fast replication time and the presence of complex viral populations (*i.e.,* quasispecies) [9–11].

**Fig. 1:**
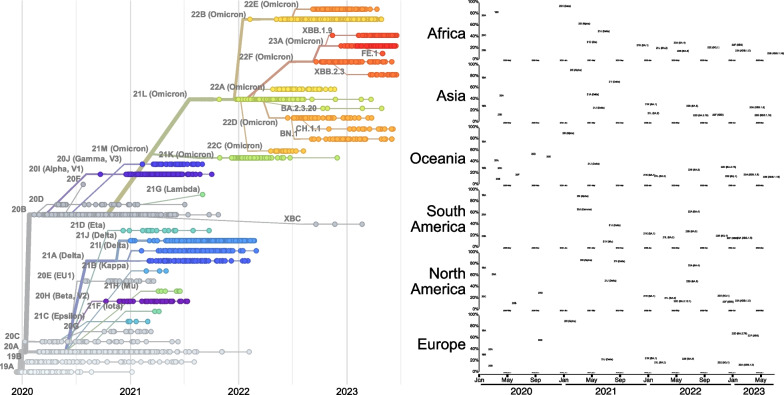
Temporal appearance of the SARS-CoV-2 strains. (Left) Each dot represents a sequenced genome at a given time point. Connecting lines indicate the evolutionary relationship among strains. The labels indicate the emerging lineages (nomenclature from nexstrain.org). (Right) Frequencies of sequenced genomes across continents (modified from nextstrain.org)

A first tier of viral diversity originates from the changes present in the many viral variants that have occurred globally (Fig. 1): starting from the first variant of concern (VOC), Alpha (B.1.1.7), initially isolated in the UK (September 2020), to Beta (B1.351, South Africa, May 2020), Gamma (P.1, Brazil, November 2020), Delta (B.1.617.2, India, October 2020). Currently circulating variants are mostly descendants of the Omicron variant (Botswana and South Africa, November 2021). The pattern of spread of variants in the first part of the pandemics, with lock-downs in place, seems rather different from what happened subsequently, when restrictions were relaxed. Indeed, up to mid-2021 several variants coexisted and had different spreading patterns across continents. As soon as travel became easier, individual variants could sweep continents in unison. While this is a testament to the infectivity of SARS-CoV-2, it is also a reminder of the importance of containment measures: while not anymore relevant to the current virulence, this is a lesson we should remember when new viral challenges will arise.

VOC-defining single-nucleotide variants (SNV) are limited and usually easily linked to fitness advantages. For example, the D614G change on the Spike protein (S) facilitates viral entry thus representing an evolutionary advantage that is conserved by positive selection in the Omicron descendent subvariants [12–14].

Another layer of complexity is revealed when intra-host SNVs (iSNVs) are considered: in single individuals the viral population is not homogeneous as iSNVs arise during viral replications in each infected cell [15–18]. This generates a mix of slightly different viruses, the so-called quasispecies. iSNVs virtually cover each position in the viral genome. While most iSNVs remain marginal, shared iSNVs become dominant, eventually determining the viral variant that will be transmitted [19, 20]. Most shared SNVs propagate through genetic drift, with many of them arising multiple times (homoplasy) in the different lineages [21–23] and only few of them will provide a gain in fitness.

Mutational and selection processes determine the evolutionary dynamics of the virus, and each of them is heavily determined by the characteristics of SARS-CoV-2.

Regarding the mutational processes, despite its size, the mutation frequency of SARS-CoV-2 is lower than that of other RNA viruses [11, 24, 25]. In fact, even though the RNA-dependent RNA polymerase (RdRp) is error-prone, similarly to that of other viruses [26], NSP14, a 3’-5’ Exonuclease (ExoN), is a proofreading element and it is fundamental in viral replication [27–29].

Beyond the intrinsic mutagenicity of the viral replication machinery, two additional mutagenic processes have been identified that result from interaction of the viral genome with the host: (i) oxidative damage, where SNVs originate from mispairing of guanines converted in 8-oxo-G adenines [30–33]; (ii) the activity of host deaminases—the APOBECs (Apolipoprotein B mRNA editing enzyme, catalytic polypeptide) and the Adenosine Deaminases Acting on RNA (ADARs) [34–36]. These enzymes convert cytidines in single-stranded RNA and adenines in double-stranded RNA into uracil and inosine, respectively.

All these mutational processes have been observed in several viruses and together they constitute the major mutagenic force that drives viral evolution [37]. In the case of SARS-CoV-2 the main host-derived source of mutations are the APOBECs, followed by oxidative damage and, to a much lower extent, by the ADARs. Both positive and negative genomic strands are targeted but, for APOBECs and oxidative damage, the positive strand is strongly preferred for the positive one [34, 38].

Finally, recombination is another factor that increases viral diversity. As in other viruses, recombination can shuffle genetic elements through exchange of segments between distinct viral genomes. In a context in which viral quasispecies abound, recombination can merge features from different coronavirus variants [39, 40].

It must be noted though that the weight of these mutational processes in viral evolution has been extrapolated by analogy with known biological processes through bioinformatic analyses on viral genomes. So far, the only host-derived mutational process whose role in SARS-CoV-2 has albeit limited experimental support is by APOBECs [41, 42].

**Fig. 2:**
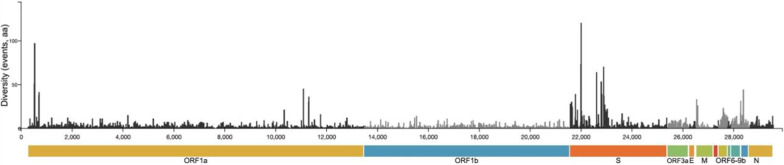
Amino acid change in SARS-CoV-2 evolution. Distribution of amino acid changes detected in viral genes, indicated in color (y axis). The number of aa changes that affect the same codon is reported on x axis (generated by nextstrain.org)

Selection is the other orthogonal force that determines evolution of the virus. The deluge of genetic and epidemiology data from the pandemic is proving once again the weight of the environment–virus–host interaction in the case of SARS-CoV-2 in the evolution of the virus. Indeed, the density of SNVs on the different open reading frames from multiple viral variants can be used to map the way the virus evolves. While there are several clusters, the gene encoding for Spike, which mediates viral entry, is the most targeted one and earlier mutations shape the potential for later ones [43]. Thus, immune pressure on the receptor-binding domain (RBD) causes convergent evolution and, eventually, immune evasion in SARS-CoV-2 variants [44]. Clusters can be similarly observed in the *ORF7*, *ORF8*, *ORF9* genes, that encode for proteins involved in immune-escape [45–47] (Fig. 2).

Beyond SNV position, there are several indicators that virus–host interaction is the main driver of viral evolution. Interestingly, long-term COVID-19 has been found to correlate with increased intra-host viral diversity [48], although we do not yet know whether it is disease persistence that drives diversity or vice versa. Analogously, iSNVs observed in chronic infections also reflect those characterizing the main VOCs [49]. An example comes from Molnupiravir (MPV), a drug approved by the FDA in 2022 for patients who “have a current diagnosis of mild-to-moderate COVID-19 and who are at high risk for progression to severe COVID-19”—the last phrase refers particularly to patients who for a variety of reasons have lowered immunity. MPV acts by inducing RNA mutations in the viral genome during the replication phase, leading to the generation of disrupted variants. However, we could imagine that some viral particles may be still viable and may be transmitted. Therefore, regardless of current debate about the extent of clinical benefits, a question about evolutionary implications is pertinent.

In favor of MPV, it does not target a specific step in viral replication so therefore, it is not expected to select for resistant mutants (like an antibiotic does). On the other hand, MPV is a potent mutagen and therefore, on top of the genetic diversity produced by spontaneous mutations and by host-induced mutations, it can produce additional genetic diversity. In principle, it is possible that one or more MPV-related mutations may confer increased infectious and/or pathogenic properties to SARS-CoV-2.

As evolutionary dynamics have changed along the course of the pandemic, epidemiological and biological factors will continue to affect viral evolution and long-term infections and zoonotic spillovers will continue to pose a threat [49–51].

Evolution mainly occurs at contact points between virus and host. Since the clash between viruses and innate immunity is subject to the whims of random mutations, we cannot share the view, or the wishful thinking recurrent in many interviews to the press, that evolutionary dynamics are oriented toward the predominance of more lenient forms of the virus. Currently in a large part of the world the virus is kept in check through vaccination and acquired immunity, but it is certainly possible that new variants with a spectrum of pathogenic potential will evolve.

### Genetic susceptibility in the host: what has changed?

Pathogen–host interactions have shaped and co-evolved over time and continue to do so today [52]. Viral infections provide strong support to this concept, because molecular adaptation to the host genome may occur very rapidly through mutations and recombination leading to an amino acid change in proteins that bind host receptors and products active in membrane fusion [53]. Therefore, not surprisingly, initial studies have been focused on the genetic variants of those genes that encode for the proteins involved in the entry of the virus into cells [54]. A recent meta-analysis evaluating 84 different studies regarding the association of 130 polymorphisms in 61 candidate genes in over 6,000 patients with severe COVID-19 and 8000 infected individuals with mild manifestations revealed a statistically significant association of ACE2 with the severity of COVID-19 [55]. The role of ACE2 was definitively confirmed through a genome-wide association study (GWAS) [56], that identified an *ACE2* variant (c.357-1203A > G, minor allele frequency 0.2–2%) that reduces by 37% (*P* = 2.7 × 10 − 8) the expression of the receptor, and therefore reduces by 40% (odds ratio = 0.60, *P* = 4.5 × 10 − 13) the risk of infection with SARS-CoV-2. Interestingly, a recent selective mapping study of SARS-CoV-2 and ACE2 revealed that SARS-CoV-2-RBD binding to hACE2 is variable. This suggests that the downregulation of additional factors such as SLC1A5, an amino acid transporter which may modulate the binding of SARS-CoV-2 to lung tissue, reduces the entry of SARS-CoV-2 variants [57].

**Fig. 3:**
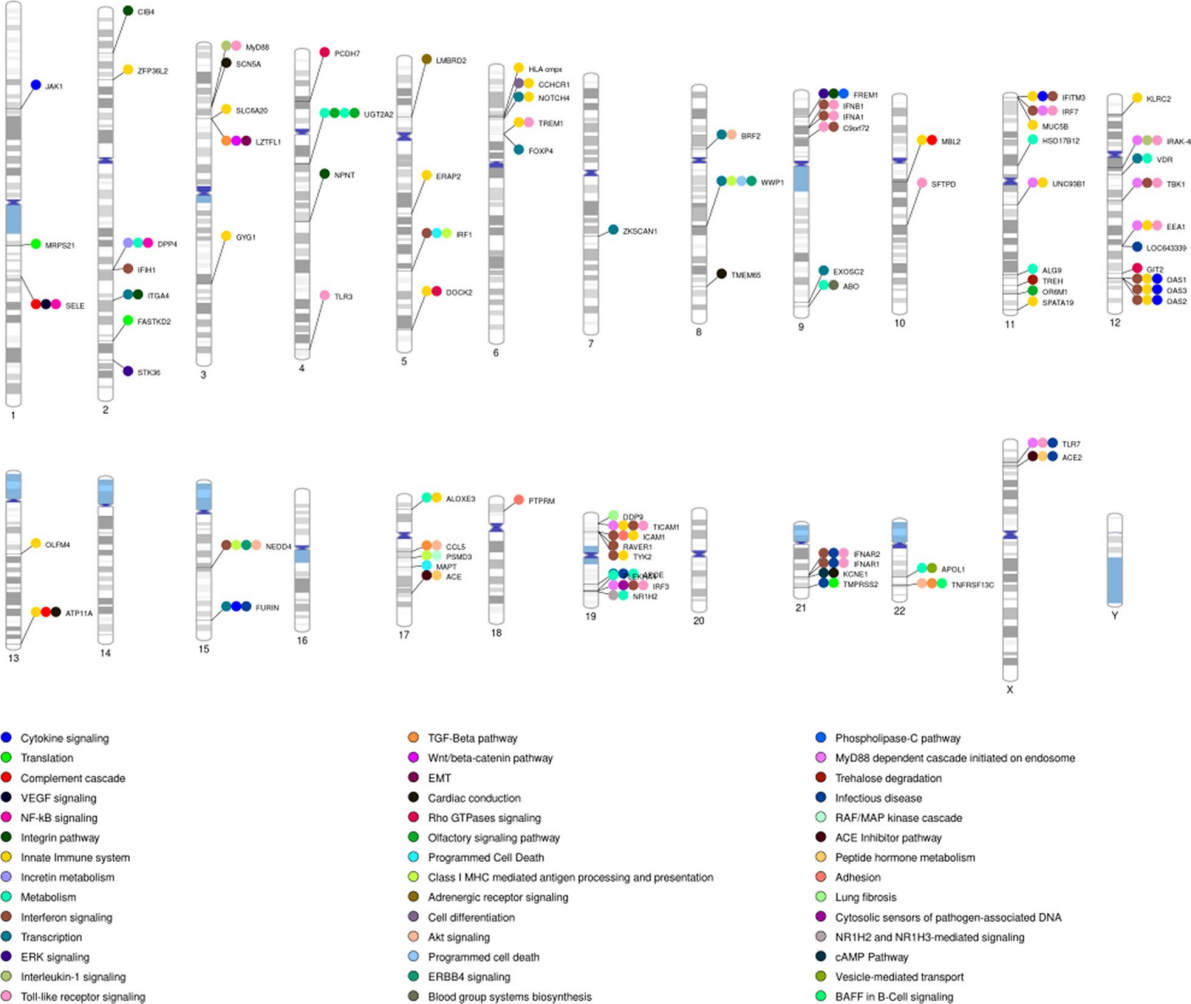
Chromosome ideogram representing the location of genes of interest investigated for a role in defining susceptibility to SARS-CoV-2 infection or COVID-19 severity (generated by visualization.ritchielab.org). For each gene, the involved pathways or mechanisms have been reported as per the legend

Several GWAS have shown and demonstrated that genetic predisposition plays a role in the susceptibility to and severity of COVID-19 [58, 59]. Significant associations were found not only with *ACE2*, but also with *SLC6A20, JAK1, IRF1, IFN-α, TLR7, DOCK2, FOXP4, SFTPD, MUC5B, CIB4, NPNT, ZKSCAN1, ATP11A, PSMD3, OAS1*. Moreover, a large European study [60] reported significant associations with *LZTF1, ABO, TYK2, MAPT, DPP9, IFNAR2*, and suggestive associations with *PCDH7, FREM1, OLFM4*, and *PTPRM* genes (Fig. 3).

A recent meta-analysis on COVID-19 severity and susceptibility to SARS-CoV-2 infection from host COVID-19 Genetic Initiative (data release 7), summarized data regarding over 200,000 cases and over 3 million controls, and identified 51 distinct significant genome-wide loci, adding 28 loci to those in a previous data release [61]. These GWAS, performed in less than two years, identified numerous candidate genes, and helped define the main biological pathways (virus entry, mucus defense, and role of interferons) involved in disease susceptibility and severity (Fig. 3). Interestingly, recent GWAS studies did not find significant associations with HLA alleles [62], contrary to what was hypothesized and expected, given the biological role of HLA in viral infections due to other coronaviruses (SARS-CoV-1 and MERS-CoV) [63–65]. The lack of association with HLA alleles of SARS-CoV-2 infection suggests that host–pathogen coevolution in balancing selection that maintains high levels of HLA allelic diversity within populations, might involve other mechanisms [66]. Furthermore, given the success of SARS-CoV-2 as a pathogen, it is not surprising that it has developed multiple strategies to evade immune responses [67]. Associations with specific Y-chromosome haplogroups have also not been found but given that Y-haplogrouping is difficult in GWAS, further targeted studies may be needed [62]. Interestingly, Augusto et al.  (Nature 10.1038/s41586-023-06331-x, 2023) revealed that the common variant HLA-B*15:01 is strongly associated with asymptomatic SARS-CoV-2 infection. About 10% of people with European ancestry have one or more copies of this genetic variant and they had a high probability of remaining asymptomatic after SARS-CoV-2 infection compared to people negative for this variant. Although this association demonstrates a "modest" odds ratio, it is still stronger than all other common variants found to date associated with COVID-19.

The candidate gene approach has also been widely used to confirm and complement GWAS-derived findings and provide useful information to identify specific pathways and proteins important in disease pathogenesis [1, 68, 69] (Fig. 3). In this regard, it is interesting that impairment of CFTR function, resulting from certain alleles of this gene, is associated with the severity of COVID-19, which also appears to activate a synergistic effect with estrogens in the response to infection [69]. Numerous other candidate genes have been analyzed and validated by functional studies to provide relevant clinical information [70, 71]. Among these, genes involved in innate immunity and those of the interferon pathway are currently regarded as important players underlying the susceptibility to SARS-CoV-2 infection and the consequent symptoms of COVID-19 [72] (Fig. 3). In fact, the cellular response to infection is initiated by viral pathogen-associated molecular patterns (PAMPs,) which are recognized by a variety of pathogen-recognition receptors (PPRs). This is followed by the activation of downstream signaling molecules such as adapter proteins MAVS and MyD88, TBK1 kinase, IKK, transcription factors IRF3, NF-κB, activator protein 1 (AP-1), and others, which results in elevated IFN-I-III production and pro-inflammatory cellular stress in general [73]. In addition, IFN-stimulated gene (ISG) expression products such as OAS, IFITM, and others seem to protect cells from SARS-CoV-2 infection by reducing virus entry [74]. Several human genetic studies carried out by the CHGE Consortium (Covid Human Genetic Effort, https://www.covidhge.com/about) have identified and characterized rare mutant alleles of these genes that confer susceptibility to COVID-19 and established causal connections between function variants of *TLR7* and *TRL3* genes and severe COVID-19 phenotype [75–77]. Remarkably, Matuozzo et al. [76] have pinpointed that the zygosity status of the mutant alleles significantly influences the penetrance of the severe disease phenotype. In this regard, it is interesting to observe how unvaccinated patients with homozygous mutations of the *MyD88* or *IRAK-4* genes have been associated with COVID-19 hypoxemic pneumonia resulting from reduced TLR7-dependent type I IFN production in plasmacytoid dendritic cells (pDCs), a subtype of immune cell otherwise known for the abundant secretion of interferon [78]. The characterization of *TLR7* variants in patients with critical COVID-19 could prove important for a possible therapeutic approach [79]. Indeed, N-acetylcysteine (NAC), through binding to TLR7 variants, may prevent NF-κB activation by scavenging ROS, inhibiting nuclear translocation of IKKb and NF-κB, and impairing pro-inflammatory cytokine synthesis [80]. Interestingly, at least five regions among the 24 identified by GWAS as critical for COVID-19 susceptibility are linked to the type I IFN pathway (TYK2, IFNAR1, OAS1, OAS2, OAS3 loci), and in 2 of them (*TYK2* and *IFNAR1*) rare predicted loss of function variants (pLOFs) have been identified in COVID-19 [75, 81, 82] (Fig. 3). However, we can note that the COVID-19 susceptibility genes, identified via GWAS and the rare variants identified through the candidate gene approach, such as those of the interferon pathway, do not always overlap. This is reminiscent of the experience in breast cancer susceptibility research, where many common variants found by GWAS in the BRCA1 and BRCA2 genes are not always the same as “rare high-risk variants” [83, 84]. It is quite evident that today, the characterization of rare variants plays a priority and unique role in the genetics of complex diseases in humans due to their distinctive characteristics, unlike the common variants. In fact, these constitute a precise objective of the functional analysis to understand the disease mechanisms, a new favorable target for the development of drugs and a valid genetic biomarker for estimating the disease risk [85, 86]. It is possible that some rare variants in GWAS genes associated with COVID-19 will be identified later when more patients are tested.

Common genetic variants have reportedly only a modest effect and explain only a very small fraction of the clinical variability [87]. For this reason, we believe that the search for rare variants that confer a stronger susceptibility to life-threatening COVID-19 should extend, perhaps to subgroups of populations and within individual families [88].

### Current COVID-19 clinical knowledge state of the art

As detailed in our previous review “*COVID-19 2022 update: transition of the pandemic to the endemic phase*” [1], COVID-19 infection produces a multi-system condition that primarily involves the respiratory system. It can lead to a systemic inflammatory response and potentially to multiorgan impairment, and death in the most critical cases [89]. Over the last two years of the pandemic, with the increase in the immunization rate in the world population, the availability of new therapies, and the spread of new variants and subvariants, the symptoms pattern has evolved in parallel. In this context, we have witnessed a mitigation of signs and symptoms, at the expense of a faster diffusion and lower lethality [90]. Despite efforts, severe outcomes cannot be eluded in all cases, even in vaccinated subjects, as recently reported in literature [91]. The ease with which currently available mRNA vaccines [92] can be updated has made it possible to deal with the new variants of the virus in a timely manner [93, 94].

Persistence of shortness of breath, exercise limitations, fatigue, neurological and vascular manifestations, are the most common of Long COVID syndrome [1]. As discussed in some detail in our previous update, this syndrome is as a multiorgan systemic condition [1], the pathophysiology of which is still under investigation [95]. Understanding the mechanisms of the infection’s sequelae may pave the way to new therapeutic perspectives [95]. We have already discussed the possibility of applying antiplatelet and antioxidant therapy in affected patients [1]. Recently, epipharyngeal abrasive therapy (EAT) for the treatment of chronic epipharyngitis post SARS-CoV-2 infection has been reported [96]. Chronic pain is the most common symptom [97, 98], and neuromuscular pain may persist for up to one year after the primary infection [99]. An interesting, randomized case–control trial has demonstrated that an endocannabinoid-like mediator (co-ultramicronized palmitoylethanolamide/luteolin) can enhance GABA-ergic transmission and reduce neuro-inflammation in a group of patients showing cognitive dysfunction (or “brain fog” [100]) and fatigue after COVID-19 [101]. Subarachnoid hemorrhage may occur in about 0.1% of COVID-19 patients, and whether this entails further neurovascular sequelae should be investigated [102].

### Brief therapeutic approach overview

On April 20, 2023, the COVID-19 Treatment Guidelines Panel published major revisions to the therapeutic recommendations [103].

Below we provide an overview of currently approved treatments.

Currently, Remdesivir is the only FDA-approved antiviral drug targeting the RNA-dependent RNA polymerase. Administered to adults and children aged ≥ 28 days, its efficacy was recently confirmed in a meta-analysis covering data from 10,480 patients, with a significant reduction in 28-day mortality in cases requiring no or low-flow oxygen administration [104]. Although more investigations are needed and against predictions due to improved immune evasion strategies, initial evidence shows that Remdesivir maintains its neutralizing capacity in vitro even against the BQ.1.1 and XBB variants [105].

Other antiviral agents have instead received Emergency Use Authorizations from the FDA. Among them, the Paxlovid. This oral formulation contains nirmatrelvir, a protease inhibitor, and ritonavir, a pharmacokinetic boosting agent that inhibits cytochrome P450 (CYP) 3A4 to increase plasma concentration of the active ingredient. It is precisely this adjuvant function that determines the need for a careful management of the prescription, since it is the basis for reported drug-drug interactions.

Another therapeutic approach under EUA is Molnupiravir, a broad-spectrum ribonucleoside prodrug of beta-D-N4-hydroxycytidine (NHC), whose use is indicated within 5 days of the onset of symptoms, if the non-hospitalized patient is at high risk of severe disease. Since it appears to have lower efficacy compared to the previously mentioned treatments [106], it is not the treatment of election. Recent studies proved that Molnupiravir might be effective against the Omicron sublineages [107, 108].

On the other hand, it is contraindicated in pregnant and lactating patients due to the antiviral mechanism, and the trials did not evaluate children (MOVe-OUT [109], PANORAMIC [110]). The NHC triphosphate used by RdRp leads to the incorporation of G or A bases, introducing mutations in the RNA product. Furthermore, the affinity between NHC and G or A in the complex is stable to the point of reducing the proofreading activity, thereby increasing the mutation rate of the polymerase leading to a lethal viral replication arrest [111]. Concerns about high mutagenicity of this antiviral and its role in the viral evolution have been described in the previous paragraph and are currently under investigation [112]. Since it could influence the rate of variability and selection of SARS-CoV-2 variants, continuous monitoring of emerging variants is active.

While there are no striking evidence supporting the effectiveness of alpha and beta interferons, proposed as treatments during the early stages of the pandemic, new studies are investigating the effect of pegylated interferon lambda (PEG-IFN lambda) and the first results have recently been published. From a TOGETHER protocol-based trial [79] on a predominantly vaccinated (83%) population of hospitalized patients, a therapeutic regimen with a single subcutaneous administration of PEG-IFN lambda within 7 days of onset showed a reduction in hospitalization and emergency observation period compared to placebo administration. Although it has limitations, this study takes into consideration symptomatic patients affected by different variants; moreover, the prevalence of a risk *OAS1* gene haplotype (AAA for rs1131454-A, rs10774671-A, and rs2660-A), which allows to stratify patients based on the probability of a positive response to therapy [113], is ensured.

Antivirals proposed on the early stage of the pandemic, such as hydroxychloroquine, chloroquine, Lopinavir/Ritonavir, and ivermectin, are no longer indicated for treatment since their use has not shown significant benefits in terms of reduction of the mortality rate or improvement of clinical status. For this reason, they are not covered in this work.

A different class of agents employed in contrasting SARS-CoV-2 is that of neutralizing antibody products. Many efforts have been made to develop monoclonal antibodies, but their effectiveness has been undermined by the appearance of resistant variants [114, 115]. From December 2022, the use of plasma from donors who have recovered from SARS-CoV-2, containing antibodies useful for the arrest of viral replication (*i.e.,* convalescent plasma), has been restricted to high titer products only, to be administered only to immunocompromised patients [116]

Immunomodulators have been used to sustain the action of antiviral agents or alone. Low-dose dexamethasone, a corticosteroid, still represents the therapeutic standard [117]. A recent randomized trial conducted on COVID-19 patients requiring simple oxygen therapy has shown that high doses of corticosteroids need to be carefully with attention as they appear to be harmful, since they increase the 28-day mortality rate [118]. The data are currently not supported by the COVIDICUS [119] and COVID STEROID 2 [120] trials, so the debate is still under investigation.

Interleukin inhibitors are also being studied, as a tool to contrast the increase in interleukins, which are associated with inflammatory damage resulting from infection and elevated in patients with COVID-19. There is insufficient evidence to recommend the use of interleukin-1 (IL-1) inhibitors, such as the recombinant IL-1 receptor agonist Anakinra, or the monoclonal antibody Canakinumab. On the other hand, studies have been conducted on inhibitors of interleukin 6 (IL-6) (RECOVERY [121], REMAP-CAP [122]), such as Tocilizumab, a repurposed monoclonal antibody. A recent update of the results obtained from the trial on the long-term effects of this therapeutic approach on critically ill patients shows a marked improvement in 180-day mortality [123]. The data are in contrast with previously conducted trials (EMPACTA [124] and REMDACTA [125]), making further investigations necessary. Another strategy to block the inflammatory cascade involves the use of Janus kinase (JAK) inhibitors, preventing the phosphorylation of proteins involved in downstream signaling (JAK-STAT pathway). Among these, promising randomized trials to have been conducted on Baricitinib, a selective inhibitor of JAK1 and JAK2 [126], and on Tofacitinib, a selective inhibitor of JAK1 and JAK3 [127].

What we have learned in these years of study and struggle in search of increasingly effective and accessible therapies is that the emergence of novel variants could compromise the work done so far. This observation should prompt us to direct future efforts toward the development of pan-coronavirus vaccines and inhibitors.

### Outlook

As the item “COVID-19” in PubMed returns, on July 3, 2023, 371,188 results, one might ask what factors have fueled this pandemic of publications regarding a viral pandemic: is it the public health burden, the scientific interest in SARS-CoV-2 evolution and in virus–host cell interactions, the clinical interest in the panoply of manifestations, the laudable re-conversion of laboratories to research on a major urgent world-wide problem, the priority funding of research on COVID-19, the enormous financial interests related to production of vaccines and of potential therapies, the jumping on the band-wagon of a topic for which journals have provided a fast-track publication pathway, possibly coupled with the publish-or-perish imperative. We daresay, all the above.

Therefore, we must perhaps justify why with this manuscript, we increase the total to 364,655. We certainly did not intend to attempt a review of this massive topic; rather, we wished to focus on some aspects for which an update may be in order.

Like in many other infectious diseases, the clinical picture is complex, since it depends on the interaction between two biological entities. After ACE2 receptor-mediated entry of SARS-CoV-2 into epithelial cells of the respiratory tract, pattern-recognition receptors and endosomal toll-like receptors (TLRs) are engaged by viral single-stranded RNA; whereupon, downstream signaling cascades trigger the secretion of type I/III interferons (IFNs), that could potentially kill virus-infected cells. However, it seems that SARS-CoV-2, like other coronaviruses, can interfere with one or more steps of this fundamental mechanism of antiviral innate immunity [128]. The resulting infection of the respiratory tract could be regarded as “regular” COVID-19: with acquired immunity setting in, this is a self-limited disease in most cases. On the other hand, severe life-threatening COVID-19 is probably related in most cases either to host fragility, or to the down-side offshoot of innate immunity, namely inflammation. SARS-CoV-2 proteins might induce IL-6 and IL-8 production, potentially by inhibiting an endogenous NF-kB repressor [129]; and many pre-disposing host factors can contribute to hyper-inflammation [130], eventually precipitating a potentially fatal cytokine storm, i.e., an adverse outcome pathway [131]. In keeping with this, several genes discussed in the above host genetic susceptibility section are part of the innate immunity network; and it is particularly interesting that rare variants of *TLR7* are associated with impaired signaling [132].

As outlined in the section on evolution and evolvability, this process has been naturally a major feature of SARS-CoV-2 since the onset of the pandemic. Random mutations are part and parcel of every round of viral replication, of which there have been zillions; and it is remarkable that mutations may result not only from errors by the RNA-dependent RNA polymerase encoded by the viral genome, but also from the same genome being edited by ADAR and APOBEC enzymes of host cell origin [33]. This finding has been challenged [34], but then validly upheld [35]. Most of the tens of thousands of SARS-CoV-2 mutations are essentially neutral; only a very small minority may undergo positive selection, as they increase the rate of transmission, and/or the severity of disease, and/or increase the risk of reinfection and reduce the protection afforded by neutralizing antibodies and vaccination [133]. The importance of tracking mutants in all parts of the world has emerged prominently through the remarkable work from the group of Tulio de Oliveira at the University of Stellenbosch in South Africa [134], that has documented the spread of several omicron variants. In the case of bacteria antibiotics have been for decades a major source of selection of resistant mutants; therefore, we have to consider how far a new antiviral agent may be similar agents of selection.

In September 2022, when asked whether the COVID-19 pandemic was finished, the Director-General of WHO Tedros Adhanom Ghebreyesus said that: “We are not there yet; but the end is in sight”. Dr Tedros further said that the world has never been in a better position to end the COVID-19 pandemic. On March 9, 2023, one of the main trackers of COVID-19, the Johns Hopkins Coronavirus Research Center, whose maps many of us have perused regularly, has decided to close down (but the wealth of existing data will remain available).

SARS-CoV-2 has not been eradicated, in the way that the smallpox virus has; however, from the almost total abrogation of physical and of travel restrictions, and especially from sensing the mood of people, it seems reasonable to say not only that the worse is over, but that COVID-19 is no longer conditioning the world. If, from now on, people at large will use common sense in aiming to avoid transmission of respiratory infections through droplets and through aerosols, including the wearing of masks when appropriate, this will be at least one positive legacy from COVID-19. Instead, a most unwelcome legacy, still very much with us, is a wide range of psychological consequences from the pandemic and from lockdown periods: both have been traumatic stressors [135], and they are still causing eating disorders and other self-damaging behavior patterns [136] that need care by professionals and by families.

## Conclusions

In several countries, there have recriminations about the fact that, in 2019, health services in most countries were not as prepared for a pandemic as they should have been. As we are not public health experts, we feel unfit to judge the perfect balance between resources in the area of prevention and containment to be in readiness for facing new contingencies, *versus* resources needed all the time for facing current medical needs. One thing is clear: namely, that the public sector of the health services has taken the brunt of the pandemic, and that things have gone better, with respect to both clinical outcomes and vaccination, in countries where a National Health Service (NHS) is in place and is efficient. It may be wise to plan and steel ourselves against another pandemic: but we suggest that the top priority is strengthening the NHS in every country that has one, and to introduce a NHS in every country that does not yet have one. There is no doubt that the world must remain vigilant about the evolution of COVID-19 and SARS-CoV-2: this requires world-wide cooperation and it is vital that coordination by the WHO remains vigorous.

Lastly, there will be ongoing progress in the human genomics of susceptibility to the disease and evolution of the virus itself. These scientific developments should be closely monitored.

## Data Availability

Data sharing is not applicable to this article as no datasets were generated or analyzed during the current study.

## Abbreviations

ADARs: Adenosine deaminases acting on RNA
APOBEC: Apolipoprotein B mRNA editing enzyme, catalytic polypeptide
CHGE: COVID human genetic effort
COVID-19: Coronavirus disease 2019
FDA: US food and drug administration
GWAS: Genome-wide association study
IFN: Interferon
ISG: Interferon-stimulated gene
iSNVs: Intra-host single-nucleotide variants
LNP: Lipid nanoparticles
MERS-CoV: Middle East respiratory syndrome-related coronavirus
MPV: Molnupiravir
NAC: N-acetylcysteine
NHS: National health service
PAMPs: Pathogen-associated molecular patterns
PRRs: Pathogen-recognition receptors
pDCs: Plasmacytoid dendritic cells
PEG-IFN lambda: Pegylated interferon lambda
pLOF: Predicted loss of function variants
RBD: Receptor-binding domain
ROS: Reactive oxygen species
SARS-CoV-1: Severe acute respiratory syndrome-related coronavirus 1
SARS-CoV-2: Severe acute respiratory syndrome-related coronavirus 2
SNV: Single-nucleotide variants
TLRs: Toll-like receptors
VAERS: Vaccine adverse event reporting system
VOC: Variant of concern
VLP: Virus-like particle
UN: United Nations
WHO: World Health Organization
